# Supplementary material for: Aluminum-Immobilizing Rhizobacteria Modulate Root Exudation and Nutrient Uptake and Increase Aluminum Tolerance of Pea Mutant E107 (brz)
Source: Plants (Basel). 2023 Jun 15;12(12):2334. doi: 10.3390/plants12122334 (PMC10304612; doi:10.3390/plants12122334)
Supplement: Supplementary file 1 [file plants-12-02334-s001.zip › plants-2400364-supplementary.pdf]

SUPPLEMENTAL MATERIAL

Table S1. Growth of *Cupriavidus* sp. D39 in batch culture supplemented with various organic compounds as a sole sources of carbon or nitrogen.

Substance	Growth using a carbon source	Substance	Growth using a carbon source	Growth using a nitrogen source
Organic acids		Amino acids		
Acetate	+++	γ-Aminobutyrate	+++	+
Citrate	+++	Alanine	+++	+
Fumarate	+++	Arginine	0	+
Indole-3-acetate	++	Asparaginate	+++	+++
Lactate	+++	Cysteine	+	+
Malate	+++	Glutamate	+++	++
Propionate	+++	Glycine	+++	+
Pyruvate	+++	Histidine	+++	++
Pyroglutamate	+++	Isoleucine	+++	+
Salicylate	+++	Leucine	+++	+
Succinate	+++	Lysine	++	+
Sugars		Methionine	0	+
Arabinose	++	Ornithine	+	+
Fructose	+++	Phenylalanine	+++	+
Glucose	++	Proline	+++	+
Ribose	++	Serine	++	+
Sucrose	++	Threonine	+++	+
		Tyrosine	+++	+
		Tryptophane	+++	++
		Valine	++	+

Note: 0, no growth; +, weak growth; ++, good growth; +++, abundant growth.

Table S2. The amount of amino acids (μg g^{-1} root dry weight) exuded by roots of cultivar Sparkle and E107 (*brz*) mutant inoculated with *Cupriavidus* sp. DG39 and treated with 80 μM $AlCl_3$.

Pea genotype and treatment	Alanine	Aspartic acid	Glutamic acid	Ornitine	Valine
		Sparkle			
Control	9.4 ± 3.1 c	16.0 ± 2.1 b	7.2 ± 3.0 a	0.05 ± 0.02 a	42 ± 8 b
Cupriavidus sp. DG39	3.0 ± 2.4 ab	1.6 ± 0.5 a	6.0 ± 0.5 a	0.01 ± 0.01 a	nd
$AlCl_3$	2.4 ± 0.6 ab	2.7 ± 0.3 a	1.5 ± 0.3 a	0.03 ± 0.02 a	8 ± 2 a
Cupriavidus sp. DG39 + $AlCl_3$	0.7 ± 0.2 a	1.2 ± 0.2 a	7.7 ± 1.5 a	0.05 ± 0.01 a	nd
		E107 (*brz*)			
Control	25.6 ± 4.6 d	14.5 ± 3.5 b	43.7 ± 7.1 b	0.08 ± 0.02 a	91 ± 28 c
Cupriavidus sp. DG39	0.2 ± 0.1 a	1.1 ± 0.1 a	2.1 ± 0.2 a	0.07 ± 0.03 a	nd
$AlCl_3$	6.6 ± 2.0 bc	21.4 ± 5.8 b	46.0 ± 11.9 b	0.21 ± 0.06 b	54 ± 16 b
Cupriavidus sp. DG39 + $AlCl_3$	0.2 ± 0.1 a	0.7 ± 0.3 a	3.1 ± 1.7 a	0.07 ± 0.03 a	nd

Note: Different lowercase letters show significant differences between treatments (least significant difference test, $p < 0.05$, n = 3). nd stands for not detected.
